# New Insights into Flexible Transparent Conductive Silver Nanowires Films

**DOI:** 10.3390/ijms20112803

**Published:** 2019-06-08

**Authors:** Yuehui Wang, Xing Yang, Dexi Du, Yuzhen Zhao, Xianfeng Zhang

**Affiliations:** 1 Department of Materials and Food, University of Electronic Science and Technology of China Zhongshan Institute, Zhongshan 528402, China; 2 School of Materials and Energy, University of Electronic Science and Technology of China, Chengdu 610054, China; shirleywyh@126.com (X.Y.); dudexi_work@foxmail.com (D.D.); 3 Department of Materials Science and Engineering, Tsinghua University, Beijing 100084, China; zhaoyz@mail.tsinghua.edu.cn

**Keywords:** silver nanowire, flexible transparent conductive film, sonication-induced scission, haze

## Abstract

Flexible transparent conductive films (FTCFs) composed of silver nanowires (AgNWs) have become an important research direction because of their potential in flexible electronic devices. The optoelectronic properties of FTCFs composed of AgNWs of different lengths were evaluated in this study. AgNWs, with an average diameter of about 25 nm and length of 15.49–3.92 μm were obtained by a sonication-induced scission process. AgNW-FTCFs were prepared on polyethylene terephthalate substrates using a Meyer bar and then dried in the ambient environment. The sheet resistance, non-uniformity factor of the sheet resistance, the root mean square roughness, and haze of the FTCFs increased as the length of AgNWs decreased. The transmittance of the films increased slightly as the length of AgNWs increased. AgNWs with a length of 15.49 μm provided an AgNW-FTCF with excellent properties including haze of 0.95%, transmittance of 93.42%, and sheet resistance of 80.15 Ω∙sq^−1^, without any additional post-treatment of the film. This work investigating the dependence of the optoelectronic properties of AgNW-FTCFs on AgNW length provides design guidelines for development of AgNW-FTCFs.

## 1. Introduction

As the demand for flexible and wearable electronic devices has increased over the last 20 years, the development of the flexible transparent conducting films (FTCFs) with excellent electrical conductivity, flexibility, and optical transparency has become important. At present, indium tin oxide (ITO) is a widely used material for the transparent conducting films; however, because of its intrinsic brittleness, relative rarity, and expensive deposition and post-treatment processes, ITO is not suitable for flexible device fabrication. To circumvent the shortcomings of ITO, many materials, including carbon nanotubes [1,2], graphene [3,4], and metal nanowires [5,6], have been studied to find appropriate materials for use as FTCFs.

Silver nanowire (AgNW) films have excellent optical and electrical properties comparable to that of ITO and are regarded as a leading candidate for FTCFs [5,6,7,8]. The properties of AgNW-FTCFs makes them attractive for usage in FTCF wearable devices, organic light emitting diodes, displays, heaters, and other flexible electronics applications [9,10,11,12,13,14,15]. An AgNW-FTCF contains a percolation network consisting of randomly distributed and overlapped AgNWs. The conductive networks of AgNW-FTCFs are only composed of AgNWs and pores, and several studies have indicated that the properties of AgNW-FTCFs are defined mainly by the size, aspect ratio, and purity of AgNWs, contact between AgNWs and network morphology [16,17,18]. Longer AgNWs may form a network with longer conducting paths, lower deposition density and a decreased number of junctions between AgNWs, which lowers junction resistance. These factors indicate that longer AgNWs can improve the conductivity of FTCFs. Meanwhile, thinner AgNWs decrease the deposition area and light scattering, improving the optical transparency and haze of FTCFs [19,20]. The high aspect ratio of AgNWs enables electrical percolation without covering the whole substrate surface [19,20]. However, the large-scale synthesis of AgNWs with long length and small diameter is still difficult.

Generally, it is challenging to fabricate a high-performance AgNWs film with both high transmittance and high electrical conductivity, because AgNWs are not transparent. Meanwhile, the haze of AgNW-FTCFs strongly depends on the diameter of AgNWs and is decreased by using AgNWs with a smaller diameter [19,20,21]. High uniformity and low haze are two importance factors for high-end applications of the AgNWs–FTCFs. However, the reported haze and uniformity of such films are still bottlenecks in current research. Chen et al. [22] prepared FTCFs using highly purified AgNWs and achieved a remarkably low haze of 0.8% and a small sheet resistance non-uniformity factor of 5.0% at 71.2 Ω∙sq^−1^. Bergin et al. recently reported that the transmittance of AgNW-FTCFs with AgNWs of a given diameter is linearly to area coverage, and does not depend on the length of the AgNWs. In addition, they found that decreasing AgNWs diameter improved optoelectronic performance only for AgNWs with a diameter of less than 20 nm [23]. Sorel et al. measured the transmittance and sheet resistance for a large number of networks of AgNWs with different lengths and diameters [24]. They found that the network direct current conductivity scaled linearly with wire length, whereas the optical conductivity remained approximately constant regardless of nanowire length. Many studies have focused on controlling the topology of AgNWs networks and AgNWs distributions [25,26,27,28]. However, very few systematic experimental studies have evaluated the relationship between the electro-optical properties and microstructure of AgNW-FTCFs.

Herein, we produce AgNWs with different lengths by a sonication-induced scission process and explained relationship of the length of AgNWs with ultrasonic time and then fabricate FTCFs composed of the AgNWs of different lengths. Furthermore, the influence of AgNWs length on the sheet resistance, non-uniformity factor of the sheet resistance, transmittance, haze, and root mean square roughness of AgNW-FTCFs were discussed in a systemic way. In addition, the relationship of the optoelectronic properties of film with microstructures was discussed. The uniformity of the AgNWs networks decreases with decreasing of AgNWs length. The resulting FTCF composed of 15.5 μm AgNWs has high transparency, low sheet resistance and haze and root mean square roughness, and high uniformity. We would like to point out that the AgNW-FTCFs conductive film prepared have no any additional post-treatment.

## 2. Results and Discussion

### 2.1. Sonication-Induced Scission of Silver Nanowires

**Figure 1:**
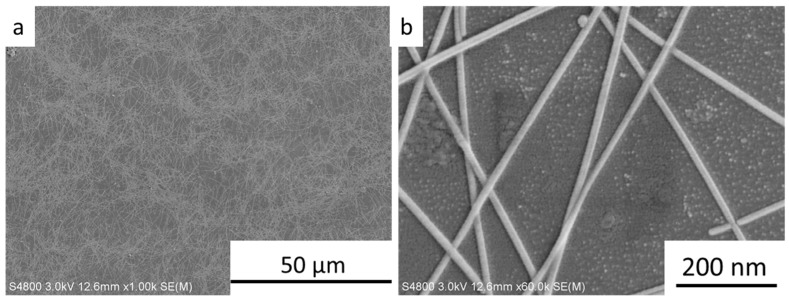
SEM images of commercial AgNWs at low (**a**) and high (**b**) magnification.

**Figure 2:**
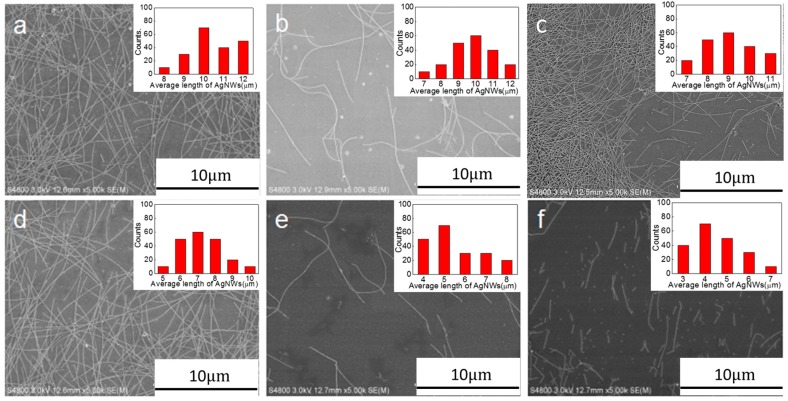
SEM images of AgNWs subjected to sonication-induced scission at an ultrasonic power of 300 W for (**a**) 0.5, (**b**) 1.0, (**c**) 1.5, (**d**) 2.0, (**e**) 2.5, and (**f**) 3 h. Insets show the corresponding AgNWs length distributions.

Figure 1a,b shows SEM images of the commercial AgNWs. The average length and diameter of the AgNWs were 25 nm and 15.49 μm, respectively. SEM images of the AgNWs (1.7 mg∙mL^−1^) after sonication-induced scission at an ultrasonic power of 300 W for 0.5, 1.0, 1.5, 2.0, 2.5, and 3.0 h, are presented in Figure 2. The inserted images display the length distribution of AgNWs in each sample. After 0.5, 1.0, 1.5, 2.0, 2.5, and 3.0 h of ultrasonic treatment, the length of the AgNWs decreased from 15.5 μm before treatment to 9.48, 9.24, 8.95, 7.86, 5.17, and 3.92 μm, respectively. AgNW scission was attributed to the sonication energy [29]. However, the change in the AgNWs diameter was not observed. The length distributions of sonicated AgNWs were considerably narrower than that of the initial length distribution of AgNWs, indicating that the long AgNWs were fragmented during ultrasonication and short AgNWs were not. This results suggestion that the decrease in AgNW length is dominated by a mechanical shearing process resulting from the fluid flow [29,30]. The length of AgNWs during sonication-induced scission decreased obviously within the first 0.5 h, over the next 2 h, the length of the AgNWs gradually decreased. The reason for this behavior is that shorter AgNWs can be dragged away more easily by the fluid flow than longer AgNWs [29]. 

**Figure 3:**
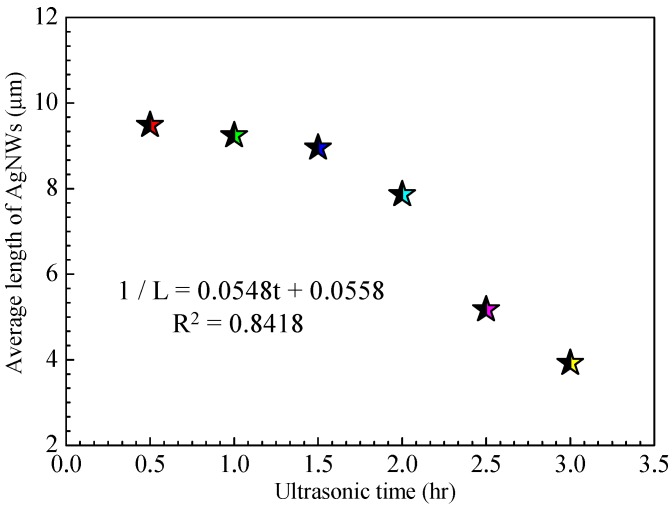
Relationship between the average length of AgNWs and ultrasonic treatment time.

Figure 3 shows the relationship between the average length of AgNWs and ultrasonic treatment time. By fitting the data points, we obtained the linear function between the average length of the AgNWs and ultrasonic treatment time, shown in Equation (1):

(1) $$1/L\;=\;0.0548t+0.0558{,\;R}^{2}=0.8418$$

where *t* is the ultrasonic time and *L*(*t*) represents the average length of AgNWs at a certain *t*. The ultrasonic energy at constant ultrasonic power is proportional to the ultrasonic treatment time. 

### 2.2. Flexible Transparent Conductive Films of Silver Nanowires

**Figure 4:**
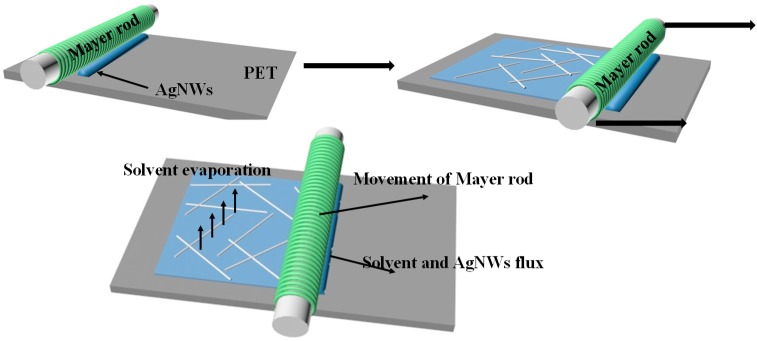
Schematic diagram illustrating the fabrication of an AgNW-flexible transparent conducting films (FTCF) using the Mayer rod method.

Conductive films consisting of AgNWs of different lengths were fabricated on polyethylene terephthalate (PET) substrates using a Meyer bar. A schematic diagram of the fabrication of an AgNW-FTCF is shown in Figure 4. Conductivity and the uniformity of conductivity of AgNW-FTCFs are important factors for evaluating the performance. Jia et al. defined a non-uniformity factor (*NUF*) to evaluate the standard deviation of the sheet resistance of the films from the average value as follows [31]:

(2) $$NUF=\sqrt{\frac{\sum_{i=1}^{n}{\left(R_{i}-\bar{R}\right)}^{2}}{n{\bar{R}}^{2}}}$$

where *n* is the number of measurements of the film of different sites, and *R_i_* and $\underset{˙}{\bar{R}}$ are the measured resistance and average resistance for all the measurements, respectively. The smaller *NUF*, the more uniform the film. According to this method, we divided film into 64 regions of the same size, measured the sheet resistance of each region and recorded the data. 

**Figure 5:**
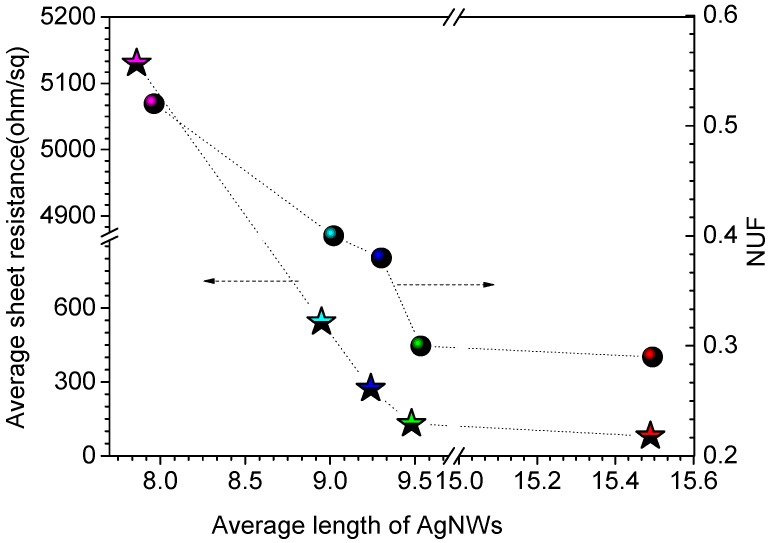
Sheet resistance and non-uniformity factor (*NUF*) of the sheet resistance of films consisting of AgNWs of different lengths.

Figure 5 presents the sheet resistance and *NUF* of the sheet resistance of films consisting of AgNWs of different lengths. Results for the films containing AgNWs with average lengths of 5.17 and 3.92 μm are not shown in Figure 5 because these films were not conductive. The sheet resistance of the lengths of AgNW-FTCFs with AgNW lengths of 15.49, 9.48, 9.24, 8.95, and 7.86 μm were 80.15, 130.23, 273.46, 543.23, and 5130 Ω∙sq^−1^, respectively. As the length of the AgNWs decreased from 15.5 to 7.86 μm, the sheet resistance of the film increases by about 6400%. As the length of the AgNWs in the AgNW-FTCFs decreased from 15.5 to 7.86 μm, *NUF* rose from 0.29 to 0.52, indicating that the distribution uniformity of AgNWs on the PET surface decreased. As the distribution uniformity of AgNWs decreases, it is difficult to AgNWs to form continuous and effective conductive paths in all direction of our films were considerably smaller than those reported in the literature [32,33], indicating that our AgNW-FTCFs have good uniformity.

**Figure 6:**
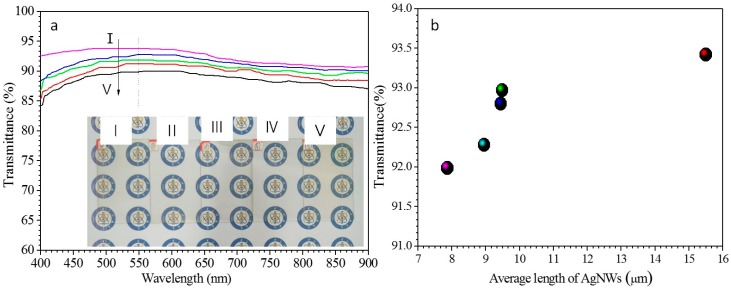
Transmittance (**a**) and transmittance at 550 nm (**b**) of AgNWs films consisting of the different lengths of AgNWs. The inset in (**a**) from I to V contains photographs of the films composed with AgNWs lengths of 15.5, 9.48, 9.24, 8.95, and 7.86 μm, respectively.

Transmittance spectra and the transmittance values at a wavelength of 550 nm of the AgNW-FTCFs containing AgNWs of different lengths are depicted in Figure 6a and b, respectively. The inset in Figure 6a contains photographs of the films. AgNW-FTCFs containing AgNWs with lengths of 15.5, 9.48, 9.24, 8.95, and 7.86 μm, displayed the transmittance values at 550 nm of 93.42%, 92.97%, 92.80%, 92.28%, and 91.99%, respectively. These values show that the transmittance decreases slightly as the length of AgNWs shortens. Comparison with Figure 5 reveals that the influence of AgNWs length on film conductivity is much greater than that on optical transparency 

**Figure 7:**
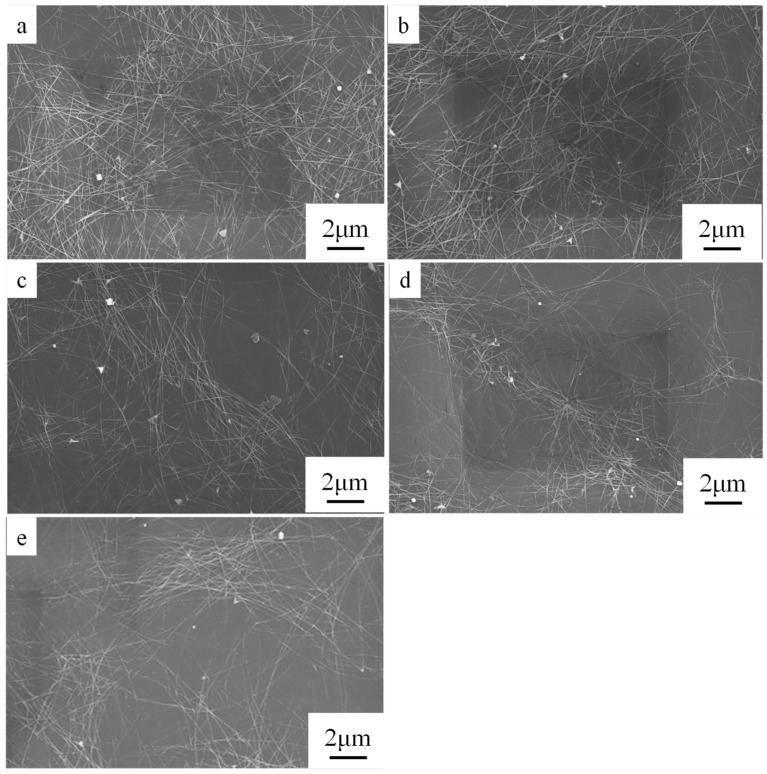
SEM images of AgNW films consisting of AgNWs with length of (**a**) 15.5 μm, (**b**) 9.48 μm, (**c**) 9.24 μm, (**d**) 8.95 μm, and (**e**) 7.86 μm.

SEM analysis provided an explanation for the influences of AgNW length of on the optical and electric performances of the AgNWs films. Figure 7 reveals that with decreasing AgNW length, the uniformity of the AgNWs distribution decreased, which might be a partial removal of PVP that leads to agglomeration of AgNWs. AgNWs with length of 15.5 μm (Figure 7a) and 9.48 μm (Figure 7b) formed continuous networks that with multiple overlapping junctions between different nanowires; the nanowires within the network were randomly or almost randomly arranged, and evenly distributed on the PET surface. Conversely, when the AgNWs with a length of 7.86 μm formed a discontinuous network on PET surface, which lead to the poor electrical conductivity of this film.

Tighter contact between crossed AgNWs resulted in higher conductivity; the long AgNWs in the network provided multiple electrical pathways from one edge of the network to the other, which meant that breaking a relatively small number of junctions would still leave alternative electrical paths from one edge of the network to the other. However, when the length of AgNWs and the distribution uniformity decrease, the discontinuous AgNWs networks are formed and the contact resistance increased considerably, resulting in a substantial decrease of electrical conductivity. In addition, for the theoretical sticks with a given length (*L*) that are widthness in two dimensions, the critical number density (*N_C_*) of sticks required for percolation is given by Equation (3) [34,35]:

*N_C_* × *L*^2^ = 5.71 (3)

**Figure 8:**
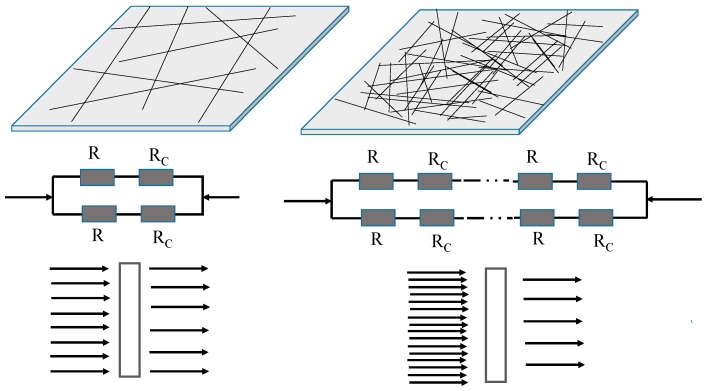
Schematic diagram of the relationship between the AgNWs length and optoelectronic properties of their thin films.

Equation (3) indicates that the *N_C_* of AgNWs required to form a percolation network is inversely proportional to the square of *L*. Therefore, a low number density of long AgNWs can form a sparse and effective percolation network. Such a network can not only increase the light transmission, but also improve conductivity forming long percolation routes with few nanowire junctions. Figure 8 illustrates the effect of AgNW length on network connectivity and thus optoelectronic properties of the AgNW films. *R_i_* is the internal resistance of AgNW and *R_c_* is the contact resistance between AgNWs. The total resistance of the conductive film can be regarded as the sum of *R_i_* and *R_c_*, where *R_c_* can be considered equivalent to the concentrated resistance, which is the resistance produced when the current flows through a very small conductive contact point and is compressed by convergence. For network structures composed of AgNWs, the number of contact points between AgNWs obviously depends on AgNW lengths. A junction resistance always exists between AgNWs. The higher the number of contact points, the higher the junction resistance in an AgNW network. In general, a typical AgNW-FTCF shows low conductivity because the AgNWs network contains numerous contact points, which leads to high junction resistance. For films composed of long AgNWs, the junction resistance is low because of the limited number of contact points. Conversely, for film composed of short AgNWs, the junction resistance is high because of the numerous contact points. This indicates that minimizing the junction resistance was important to obtain AgNW-FTCFs with high conductivity without post-treatment. 

For a given concentration of AgNWs, decreasing the length of AgNWs increases the number, which results in increased contact resistance of their film. In addition, with the decreasing AgNW lengths, the distribution uniformity of AgNWs on the substrate surface decreased, resulting in difficulty forming an effective conductive network between AgNWs, which increased the film resistance. Meanwhile, increasing the number of AgNWs increased their deposition density on the substrate, which decreased the transmittance. The effect of AgNW length on optoelectronic properties is especially important at high transmittance (low area coverage), where there are relatively few connections between nanowires, i.e., the film is porous.

**Figure 9:**
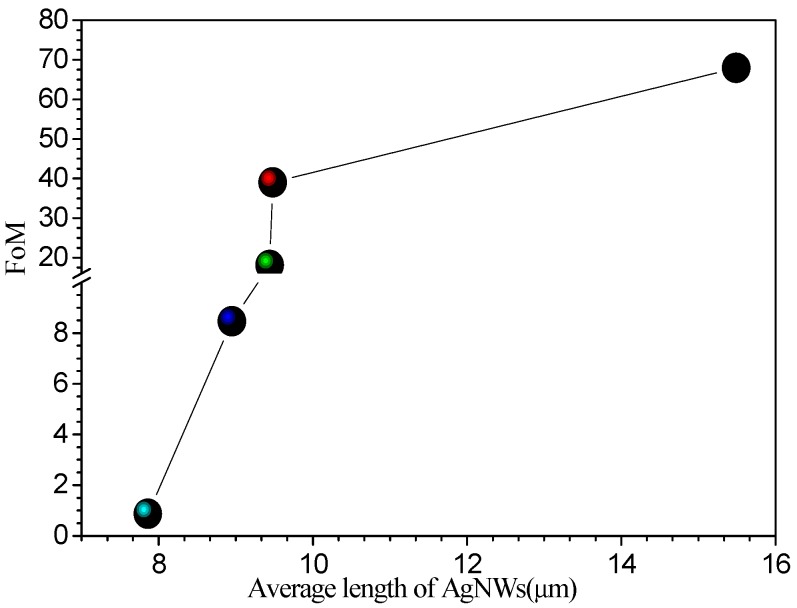
Relationship between figure of merit (FoM) of AgNW-FTCFs and the average AgNW length.

In general, figure of merit (*FoM*) is defined the ratio *σ_DC_*/*σ_Op_*, where *σ_Op_* (*λ*) is the optical conductivity and *σ_DC_* is the direct current (*DC*) conductivity of the film, which is one of the important parameters for evaluating the optoelectronic properties of optoelectronic films. Here, we calculated *FoM* of the film, as shown in Figure 9. As can be seen from Figure 9, with decreasing of AgNW length, *FoM* decreased gradually. When the AgNW length decreased from 15.5 m to 7.86 m, the film *FoM* decreased from 67.9 to 0.86, which was reduced by about 790%. This result indicated that the AgNW length has great influence on optoelectronic properties of film. As we know that transparent conductive film as electrode to be used in optoelectronic devices, the *σ_DC_*/*σ_Op_* value is at least 35, which corresponds to the resistance sheet less than 100 Ω with a transmittance at 550 nm over 90%, meanwhile, *σ_DC_*/*σ_Op_* values of film need over 50 and 10 when they are used in liquid crystal display and touch screen panels, respectively [36]. We would like to point out that the AgNW conductive film prepared in our lab is suitable for the above applications, especially for the display screen due to the low haze.

**Figure 10:**
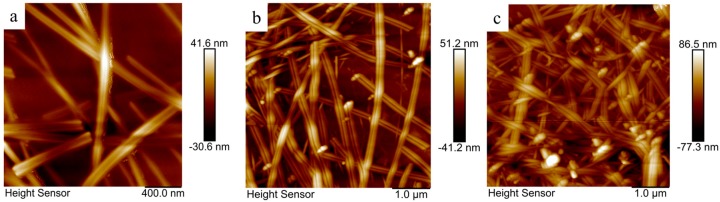
AFM images of films with AgNWs lengths of (**a**) 15.5 μm, (**b**) 9.24 μm, and (**c**) 7.86 μm.

To further study the effect of AgNW length on morphology, the films with AgNW lengths of 15.5, 9.24, and 7.86 μm were analyzed by AFM as shown in Figure 10. Theg measured root mean square roughness (*RMS*) values of the film with AgNW lengths of 15.5, 9.24, and 7.86 μm were 12.9, 18.4, and 22.3 nm, respectively. These RMS values are low approximately the diameter of a nanowire. The *RMS* values of the films increases with decreasing AgNW length, which may be related to the non-uniform distribution and high local deposition density of AgNWs.

**Figure 11:**
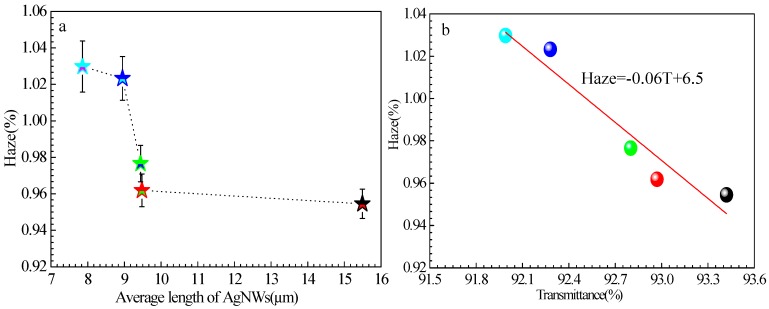
Relationship between the haze of AgNW-FTCFs and (**a**) the average AgNWs length and (**b**) transmittance at 550 nm.

Haze is defined as the ratio of diffuse transmittance to total transmittance according to ISO 14782 [37,38]. Figure 11a,b show the relationships between the haze of the film and the average AgNWs length of and film transmittance at 550 nm, respectively. The haze increases in the range 0.95–1.03% with decreasing in AgNWs length from 15.49 to 7.86 μm; these haze values are lower than those reported in the literature [19] and identical to that of ITO films. This was attributed to the smaller diameter of AgNWs used in our experiments and low RMS values of the films, which suppressed light scattering. Comparing the haze of films of AgNWs with the same diameter and different lengths revealed that haze slightly increased with e decreasing AgNW length, which may be caused by the increasing deposition density of AgNWs on the substrate and number of contact points between AgNWs. This trend is consistent with the results of reported theoretical and experimental investigations [37]. It is worth noting that the film consisting of AgNWs with a length of 15.5 μm exhibited haze of 0.95%, transmittance of 93.42%, and sheet resistance of 80.15 Ω∙sq^−1^ without post-treatment. This performance is acceptable for use of the film in display devices. 

After linear fitting of the data points plotted on a logarithmic scale, the following relationship between the haze and transmittance was found:

Haze = −0.06*T* + 6.5 (4)

**Figure 12:**
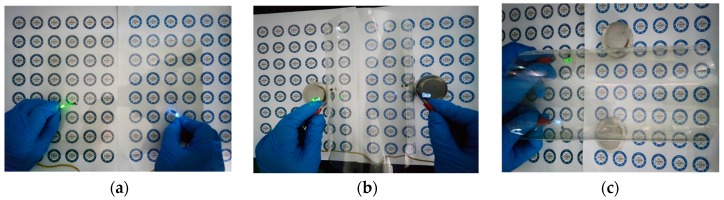
Photographs of devices containing green and blue LEDs the AgNWs film operating when (**a**) flat, (**b**) bent outward, and (**c**) bent inward.

To demonstrate the applicability of the AgNWfilms, we connected green and blue LEDs to the conductive film, as shown in Figure 12. Figure 12a–c present photographsin which the device is flat, bent outward, and bent inward. In each case, the two LEDs works normally regardless of the bending state of the conductive AgNW film.

## 3. Materials and Methods

### 3.1. Materials

An AgNW suspension was purchased from Suzhou Gushi New Materials Co., Ltd., Suzhou, China. The suspension contained AgNWs with an average diameter and length of 25 nm and 15.49 μm, respectively, dispersed in isopropyl alcohol at a concentration of 10 mg∙mL^−1^. Polyethylene terephthalate (PET) substrates were purchased from Hefei Microcrystalline Materials Co., Ltd., China. Ethanol absolute (99.7%) was purchased from Jinan Liyang Chemical Co., Jinan, Ltd., China. All the chemicals were used as received. 

### 3.2. Sonication-Induced Scission of Silver Nanowires

For ultrasonication treatments, the AgNWs suspension was diluted to 2 mg∙mL^−1^ with isopropyl alcohol and then subjected to ultrasonication for 0.5–3 h at a power of 300 W. The ultrasonication was carried out in a bath-type sonicator (JP-120ST, 0–600 W, 28/40 kHz, Shenzhen Jiemeng Cleaning Equipment Co., Ltd., Shenzhen, China). 

### 3.3. Fabrication of Flexible Transparent Conductive Silver Nanowires Film

AgNWs films were fabricated on PET substrates using a Mayer bar (No.7, R.D. Specialties Co., Ltd., Dallas, Texas, USA) and AgNWs suspension (1.7 mg∙mL^−1^) films were then dried in the ambient environment for 15 min. 

### 3.4. Characterization

The size of the AgNWs was statistically characterized by scanning electron microscope (SEM; Zeiss Sigma 500, Carl Zeiss, Oberkochen, Germany). The lengths of individual AgNWs visible in the images were measured manually using image processing software (ImageJ, version 1.38, National Institutes of Health Bethesda, MD, USA). For one sample, we obtained six SEM images. The lengths of individual AgNW in the SEM image were measured manually according to an image processing software. The number of AgNW measured for each sample should not be less than 80. The sheet resistances of films were characterized using a four-probe system (ST2253, Suzhou Jingge Electronic Co., Ltd., Suzhou, China) and optical transmittances were measured using a thin film transmittance meter (GZ502A, Shanghai Guangzhao Optoelectronic Technology Co., Ltd., Shanghai, China). Diffuse reflectance was measured with an ultraviolet-visible spectrophotometer (760CRT, Shimadzu Ltd., Kuwabaracho, Japan). Optical transmittance and sheet resistance of AgNW-FTCFs were measured at 20 different sites, from which average values were calculated. The transmission and diffuse reflectance were measuredusing a PET film as a reference. The surface morphology was analyzed via atomic force microscopy (Dimension Edge, Bruker, Billerica, MA, USA).

## 4. Conclusions

AgNWs with an average diameter of about 25 nm and a length of 3.92–15.49 μm were obtained by sonication-induced scission. AgNW-FTCFs were then prepared on PET substrates and dried in the ambient environment. The film contains AgNWs with lengths of 5.17 and 3.92 μm were non-conductive because of the poor contact between the AgNWs. The sheet resistance and *NUF* of the AgNW-FTCFs increased s by about 6400% and 178%, respectively, as the AgNWs length decreased from 15.5 to 7.86 μm. These results indicated that the distribution uniformity of AgNWs on the PET surface o decreased as the ANW length shortened. The transmittance of the film at 500 nm decreased slightly from 93.42% to 91.99% and haze increased from 0.95% to 1.03% as the AgNWs length decreased from 15.49 to 7.86 μm. RMS values of the films were low (close to the diameter of a nanowire). The film consisting of AgNWs with a length of 15.5 μm exhibited haze of 0.95%, transmittance of 93.42%, and sheet resistance of 80.15 Ω∙sq^−1^ without any additional post-treatment. These highly uniform and mechanically stable AgNW-FTCFs meet the requirement for numerous applications and could soon play a major role in the display market
